# HIV-1 Entry, Inhibitors, and Resistance

**DOI:** 10.3390/v2051069

**Published:** 2010-04-29

**Authors:** Michael A. Lobritz, Annette N. Ratcliff, Eric J. Arts

**Affiliations:** Department of Molecular Biology and Microbiology and Division of Infectious Diseases, Department of Medicine, Case Western Reserve University, 10900 Euclid Ave., Cleveland, OH 44106, USA; E-Mails: michael.lobritz@gmail.com (M.A.L.); anr16@case.edu (A.N.R.)

**Keywords:** HIV-1, envelope, gp120, V3 loop, gp41, CCR5, maraviroc, vicriviroc

## Abstract

Entry inhibitors represent a new class of antiretroviral agents for the treatment of infection with HIV-1. While resistance to other HIV drug classes has been well described, resistance to this new class is still ill defined despite considerable clinical use. Several potential mechanisms have been proposed: tropism switching (utilization of CXCR4 instead of CCR5 for entry), increased affinity for the coreceptor, increased rate of virus entry into host cells, and utilization of inhibitor-bound receptor for entry. In this review we will address the development of attachment, fusion, and coreceptor entry inhibitors and explore recent studies describing potential mechanisms of resistance.

## 1. Overview of HIV-1 Antiretroviral Therapy

The major approach to the medical management of HIV infection is the treatment of patients with antiviral drugs. The enzymatic processes of the HIV-1 replication cycle present unique approaches for targeted disruption by pharmacological agents. Due to the high rates of virus production and the mutation rate of the virus [1], treatment of HIV-1 infection generally includes administration of three agents in combination, referred to as highly active antiretroviral therapy (HAART). Sustained treatment of patients with three active drugs results in suppression of viral replication in peripheral blood to below detection limits of sensitive clinical assays (<50 RNA copies/ml). Continued virologic suppression has led to dramatic increases in the life expectancy of HIV-infected individuals and in time to diagnosis with AIDS, and decreases in HIV-associated morbidity and opportunistic infection [2]. To date, 24 individual drugs have been approved by the United States Food and Drug Administration for the treatment of HIV infection. These drugs are distributed into six major classes: 1) Nucleoside-analog reverse transcriptase inhibitors (NRTI), 2) Non-nucleoside reverse transcriptase inhibitors (NNRTI), 3) Protease inhibitors (PI), 4) Fusion inhibitors, 5) Entry Inhibitors - Coreceptor Antagonists, and 6) Integrase inhibitors (Table 1).

## 2. HIV-1 Envelope and Host Cell Entry

**Figure 1.**
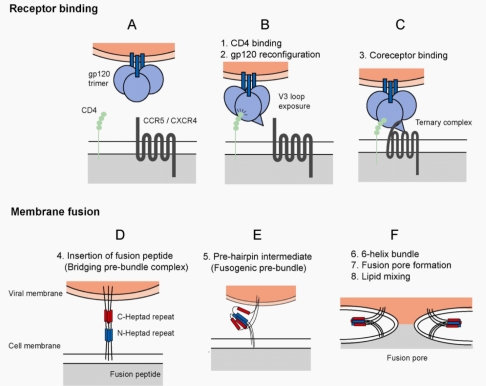
HIV-1 Entry. **(a)** Virus entry into host cells is mediated by the envelope glycoprotein. The major events of the entry process are divided into receptor binding and membrane fusion events. The functional unit of envelope is a trimer composed of three gp41 molecules (blue bars) and three gp120 molecules (blue spheres), which are associated by non-covalent interactions. **(b)** Virus entry is initiated by the attachment event, binding of gp120 to host cell CD4 (green spheres). Binding to CD4 results in reconfiguration of the gp120 molecule. The bridging sheet is formed and gp120 is primed for interaction with a coreceptor molecule. **(c)** CD4-bound gp120 interacts with a coreceptor, either CCR5 or CXCR4. The stoichiometry of the ternary complex is 1:1:1 (gp120:CD4:Coreceptor), though the number of trimer subunits and number of trimers required to initiate membrane fusion is unclear. **(d)** After interaction with the coreceptor, a hydrophobic gp41-derived peptide (the fusion peptide) is inserted into the host cell membrane. (HIV-1 gp120, CD4 molecules, and coreceptor molecules have been removed for simplification, and to allow focus on the transition in gp41 during fusion) This event anchors the virus to the host cell, and the gp41 molecule acts as a bridge between the viral and cellular membranes. Structural rearrangements within the gp41 molecule are mediated by two triple stranded coiled-coils, the C-terminal (red) and N-terminal (blue) heptad repeat domains. **(e)** Metastable prefusion intermediates occur during the reconfiguration of the gp41 domains. **(f)** The C- and N-terminal heptad repeat regions pack into one another forming a stable six-helix bundle. This results in a close approximation of viral and host cell membrane and the formation of a fusion pore. The initial fusion pore enlarges as the membrane lipids mix, ultimately leading to a fusion pore of critical size for release of the viral core into the cytoplasm.

HIV-1 can productively infect cells that express two receptors: the principal receptor CD4 [4] and an auxiliary co-receptor, which derives from the chemokine receptor family, typically either CCR5 or CXCR4 [5] (Figure 1). HIV-1 enters cells through a pH-independent membrane fusion event [6], which results in release of the core particle into the cytoplasm. However, recent studies now describe HIV-1 entry and membrane fusion following endocytosis [7,8]. The principal virus protein involved in entry is the envelope glycoprotein. The envelope gene encodes a protein that measures 160 kDa when fully glycosylated and is divided into two regions: the surface unit gp120 and the transmembrane region gp41 [9]. The gene is translated as a full length gp160 precursor protein and undergoes secondary structure formation and glycosylation in the endoplasmic reticulum [10]. Gp160 monomers then oligomerize into trimers [11] via noncovalent interactions between gp41 subunits. Processing of the protein in the golgi apparatus results in cleavage of the 160 kDa protein by members of the furin family of endoproteases into the gp120 and gp41 subunits [12]. The gp120 surface unit remains noncovalently associated with its gp41 partner [13]. The envelope is transported to the cell surface and is incorporated into virions budding from the plasma membrane.

When interacting with a new target cell, gp120 initially makes contact with the N-terminus of CD4 [14] through interactions with a conserved binding site [15] (Figure 1B). Interaction with CD4 results in considerable reconfiguration of the gp120 molecule which results in exposure of a highly conserved coreceptor binding site [16–20] (Figure 1C). The coreceptor binding site interacts with the N-terminus of a coreceptor, either CCR5 or CXCR4 (Figure 1C). Further coreceptor interactions are mediated by amino acids at the crown of the V3 loop. Ultimately, conformational changes in the envelope glycoprotein expose the fusion peptide, a sequence of 15 hydrophobic amino acids located on the N-terminus of gp41, which insert into and destabilize the host cell membrane [21,22]. At this stage, gp41 is an integral component common to the viral envelope and host membrane (Figure 1D). However, further structural transitions in gp41 are necessary to provide the significant change in free energy required to drive membrane fusion [23–25]. Prior to fusion, gp41 folds back on itself forming a hairpin structure, the function of which is to bring into close proximity the fusion peptide associated cellular membrane and the integral gp41 associated viral membrane (Figure 1E). This process is mediated by two helical regions in the ectodomain of gp41 termed HR1 and HR2 [26–28]. The N-terminal heptad repeat region, or HR1, and the C-terminal heptad repeat region, or HR2, form a stable 6-helix bundle when the antiparallel HR2 domain binds on the outer grooves of the triple-stranded coiled-coil HR1 domain, resulting in the localization of the fusion peptide and transmembrane domains at the same end of the molecule [29,30] (Figure 1F). This re-orientation and release of free energy drives the fusion of the viral and host cell membranes.

## 3. HIV-1 Receptors and Implications of Tropism

The concept of inhibiting HIV-1 replication by preventing the virus from entering the host cell has been contemplated since the first identification of the major receptor, CD4 [4,14]. Early approaches suggested the use of monoclonal antibodies to CD4 to block binding by virus. This approach was limited due to the importance of CD4 in basic immunologic functions. The next major advancement in inhibition of HIV-1 entry was the development of a soluble form of CD4 (sCD4) [31–35], which could inhibit HIV-1 replication *in vitro* and *in vivo* [36,37], as well as a soluble CD4-immunoglobulin fusion protein [38]. However, laboratory strains were significantly more sensitive to sCD4 neutralization than primary HIV-1 isolates. These differences were based on affinity and association rates for CD4 of the envelope glycoprotein quaternary structure [39,40]. In some cases, treatment with sCD4 resulted in enhancement of infection [2]. Ultimately it was observed that therapeutic administration of sCD4 had no effect on viremia or disease [41,42]; however, the sCD4 molecule provided a tool for greater understanding of the process of HIV-1 entry.

Discovery of the coreceptors that mediate HIV-1 entry was facilitated by studies showing that replication of virus could be blocked by then unknown, leukocyte derived, soluble suppressor factors [43]. The soluble factors derived from CD8^+^ T cells were identified as the C-C chemokines RANTES (CCL5), MIP-1α (CCL3), and MIP-1β (CCL4) [44]. Chemokines are small paracrine signaling molecules that are principally involved in the inflammatory response. There are currently four main classes of chemokines, and their nomenclature is based on the number and orientation of N-terminal cysteine motifs [45]. C chemokines have a single cysteine residue. C-C chemokines, C-X-C chemokines, and C-X3-C chemokines each have two cysteine residues, separated by 0, 1, or 3 other residues, respectively. Only the C-C chemokines and C-X-C chemokines are major factors in HIV-1 infection.

In 1996 the “fusin” cofactor was identified by expression of a cDNA library derived from T-tropic virus-permissive cells against a nonpermissive cell line [46]. This receptor was later identified as C-X-C chemokine receptor 4 (CXCR4), and its ligands [stromal derived factor-1 α/β (SDF-1α/β, CXCL12)] can inhibit HIV-1 replication *in vitro* [47,48]. Shortly thereafter, C-C chemokine receptor 5 (CCR5) was identified as the major entry cofactor of M-tropic, NSI HIV-1 isolates [49–53]. The chemokine receptors are members of the seven transmembrane G protein-coupled receptor superfamily. They are defined by their coupling to the pertussis toxin-sensitive G_i_ class of G proteins, expression in leukocytes, and chemotactic signaling function, and are primarily involved in leukocyte activation and directional migration. The chemokine system is highly redundant, with each receptor capable of binding multiple ligands, and each ligand promiscuously binding to multiple receptors. This same promiscuity has been investigated for the HIV-1 envelope, and it was revealed that the chemokine receptors CCR2b, CCR3, CCR7, CCR8, STRL33/BONZO, and gpr15/BOB can mediate infection of cells by some viruses [54–58]. Use of these alternative coreceptors appears limited to expression on transfected cell lines, and most evidence suggest that the receptors CCR5 and CXCR4 are the most relevant receptors *in vivo.* Currently, viruses that utilize CCR5 as an entry cofactor are referred to as R5 viruses, while viruses that utilize CXCR4 are referred to as X4 viruses [59]. Viruses that can utilize either CCR5 or CXCR4 as an entry cofactor are referred to as dual tropic, or R5X4.

CCR5-tropism is characteristic of viral isolates that persist during asymptomatic disease, and are further thought to be the principal subset of virus responsible for new infections. Over the course of HIV infection, a switch to primarily CXCR4-tropic or dual tropic isolates is generally associated with a rapid depletion of CD4^+^ T cells and progression to AIDS [60–62]. A subset of individuals at high risk for infection with HIV-1 remains seronegative despite multiple opportunities for virus transmission. Genetic analysis of these cohorts revealed that a subset of these individuals was homozygous for a 32 bp deletion in the CCR5 open reading frame, and that their CD4^+^ T cells were resistant to infection by R5 viruses *ex vivo* [63–68]. This deletion (Δ32) results in a truncated receptor that is not expressed on the cell surface. The Δ32 allele is present in the Caucasian population, with as many as 20% of Caucasians heterozygous for the mutation (*Δ32/wt)* and 1% homozygous (*Δ32/Δ32)* [63]. While individuals homozygous for the Δ32 allele are highly resistant to acquisition of HIV-1 infection (transmission of X4 viruses in *Δ32/Δ32* individuals has been reported), heterozygous individuals typically have a more protracted course of infection and experience longer time intervals before progression to AIDS. Single nucleotide polymorphisms within the promotor region of CCR5 have also been associated with differences in disease progression rates. Specifically, individuals who are – *2459A/A* have been shown to progress to AIDS more rapidly than individuals homozygous for the guanine allele (*–2459G/G)* [69–72]. Remarkably, individuals carrying these receptor polymorphisms lack any discernable biological phenotype other than resistance to HIV infection or delayed progression to AIDS, which indicated the potential value of targeting entry through the CCR5 coreceptor as a viable pharmacological intervention. The origin of the Δ32 deletion in human or primate evolution is unknown but the impact of this polymorphism is differential depending on pathogen. The Δ32 mutation has been shown to be protective against viral persistence with hepatitis B virus infection [73]. In contrast, heterozygotes and homozygotes for the Δ32 CCR5 deletion are at higher risk of early and rapid disease progression upon West Nile virus infection [74].

## 4. Interaction of HIV-1 Envelope and CCR5

**Figure 2.**
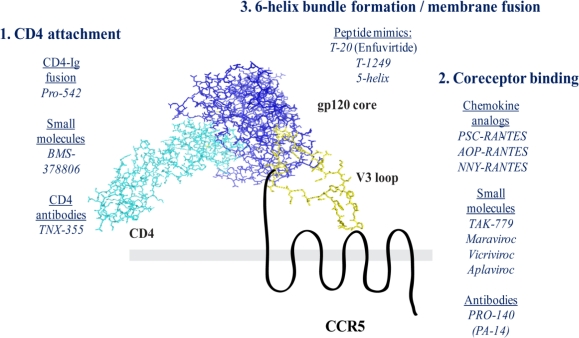
Model of Ternary Complex Formation. The model is based on the crystal structure of a V3 loop-containing structure of gp120, bound to a soluble fragment of CD4 [81]. The gp120 core (blue) is bound to the first Ig-like domain of CD4 (cyan). This interaction stabilizes the core molecule bridging sheet domain, which is composed of a series of antiparallel β-sheets. The V3 loop (yellow) extends outward from the gp120 core inner domain. The V3 crown is thought to mediate interaction with the second extracellular loop of CCR5 (black). The CCR5 N-terminus is thought to contact both the bridging sheet domain as well as the V3 base.

The envelope glycoprotein, when unligated with CD4, is not in a conformation that can interact with the coreceptor. A subset of HIV-2 viruses and SIV isolates can bind to coreceptor independently of CD4, which has led to the suggestion that the chemokine receptors are the ancestral receptors of the early lineage viruses that gave rise to modern primate immunodeficiency viruses. Use of CD4 is thought to be an adaptation to hide the conserved coreceptor binding site from recognition by the host humoral response. When gp120 binds CD4, structural rearrangements in the envelope glycoprotein lead to exposure of the V3 loop and the formation of the bridging sheet. The V3 loop is a highly heterogeneous, loop-like structure within gp120. The V3 loop is the immunodominant domain of the HIV-1 envelope, though it is not exposed on most primary isolates and is therefore not considered a major neutralization determinant [75]. The V3 loop is topographically divided into three subsections (Figure 2): 1) The base, formed by a disulfide bond and in close approximation with the gp120 core; 2) the stem, which is a flexible linker region between the base and the tip; 3) the crown, which contains a helix-turn-helix motif defined by a G-P-G-X sequence, and believed to be a critical interacting face with the extracellular loops of CCR5. V3 is the critical determinant of coreceptor tropism. As few as two amino acid changes in the V3 loop can alter the coreceptor specificity from CCR5 to CXCR4 [75]. Furthermore, changes in the V3 have been specifically associated with changes in susceptibility to entry inhibitors [75]. In addition to the V3 loop, the V1/V2 regions of gp120 have been shown to direct co-receptor activity and influence co-receptor affinity [76].

The current model of gp120-CD4-CCR5 ternary complex formation favors multiple interactions between gp120 and CCR5 (Figure 2). Studies using deletions and chimeric receptors (using parts of human CCR2b or murine CCR5, which do not mediate HIV-1 entry) suggest that the N-terminus of CCR5 is significant for HIV-1 entry [16,77,78]. Site directed mutagenesis of the V3 region indicated that two domains in gp120 were essential to the interaction with CCR5. The V3 tip, or crown, is suggested to interact with the extracellular loops of CCR5, specifically extracellular loop 2 [79]. Consistent with this, monoclonal antibodies that recognize CCR5 ECL2 potently inhibit HIV-1 entry. Post-translational modification of CCR5 plays a significant role in coreceptor activity. The N-terminus undergoes both O-glycosylation and tyrosine sulfation. Inhibition of tyrosine sulfation pathways decreases CCR5 binding by gp120 and HIV-1 entry [80]. Mutagenesis or deletion of the intracellular carboxy-terminal domain of CCR5, which mediates ligand-induced endocytosis, reveals that this is not an essential function of viral entry. Similarly, deletion or mutagenesis of the second intracellular loop DRY signaling motif indicates that intracellular signaling is dispensable for productive viral entry.

Seven transmembrane G protein-coupled receptors exist in multiple allosteric states due to their function in transmitting information from a single ligand binding site to other, topographically distinct locales, which include sites of G protein association, oligomerization, and signal transduction scaffolding protein binding [82]. As such, CCR5 exists in multiple antigenic configurations [83]. Distinct conformational states of CCR5 in either active or inactive states have been observed using the binding properties of various antibodies [83,84]. These conformational states exist transiently, and the stability of each conformation depends upon the lipid environment, the activation state of the receptor, and the ligand that is bound. Environmental stimuli can stabilize the receptor in particular antigenic configurations. Stabilization may potentially occur through receptor association with scaffolding proteins involved in signal transduction. Some reports suggest that CCR5 dimerizes through interactions in the first transmembrane region, and that the dimerized form of the molecule is nonpermissive for HIV-1 entry [85]. Dimerization of CCR5 has been suggested to be ligand-dependent, though some studies have demonstrated ligand-independent oligomerization of CCR5 [86]. The role of CCR5 conformational heterogeneity in HIV-1 entry and coreceptor interaction remains unclear at this time. It remains unknown whether HIV-1 envelope can interact with all, or only a subset of the available CCR5 conformational isomers.

## 5. Inhibition of HIV-1 Entry

The major enzymatic processes of HIV-1 replication are reverse transcription, integration, and protease maturation. Each represents a unique, virus specific event that can be targeted by drugs to block virus replication. Uncovering the major mechanisms of HIV-1 host cell entry revealed a series of critical processes that could be targeted for disruption pharmacologically. HIV-1 entry inhibitors fall into three major classes based on the specific entry process that they target: 1) attachment inhibitors, which block the interaction between HIV-1 envelope and CD4, 2) coreceptor inhibitors, which block the interaction between HIV-1 envelope and CCR5 or CXCR4, and 3) fusion inhibitors, which prevent the virus from mixing its membrane with the host cell membrane and releasing the viral core into the cytoplasm (Table 2).

### 5.1. Attachment Inhibitors

The attachment inhibitor BMS-378806 inhibits both R5- and X4-tropic HIV-1 isolates [87]. This compound binds to a pocket on gp120 important for binding CD4 and alters the conformation of the protein such that it cannot recognize CD4 [88]. The inhibitor binds with significantly higher affinity than CD4, and is an excellent candidate for therapeutic use. TNX-355 is a humanized anti-CD4 monoclonal antibody that binds to CD4 and inhibits HIV-1 envelope docking, but does not inhibit CD4 function in immunological contexts. Currently no attachment inhibitors are approved for use in patients with HIV-1 infection and none appear to be approaching phase I clinical trials.

### 5.2. Fusion Inhibitors and Mechanisms of Resistance

The crystal structure of the gp41 ectodomain [29] and of the ectodomain partnered with an inhibitory peptide (C34) [89] revealed that the fusion active conformation of gp41 was a six-helix bundle in which three N-helices form an interior, trimeric coiled-coil onto which three antiparallel C-helices pack. Enveloped viruses use a generally conserved mechanism to initiate the fusion event between viral and host cell membranes, and this six-helix bundle formation has been well studied in the paramyxoviruses family of viruses [10,90]. Peptide fusion inhibitors were designed based on the discovery that two homologous domains in the viral gp41 protein must interact with each other to promote fusion, and that mimicry of one of these domains by a heterologous protein can bind and disrupt the intramolecular interactions of the virus protein. Alpha-helical peptides homologous to the leucine zipper domain of gp41 had significant antiviral activity against HIV-1, and this activity depended upon their ordered solution structure [91]. Rational design ultimately produced a molecule (T-20, enfuvirtide) with potent antiviral activity *in vivo* [92,93], and many other peptide mimics have been described (Table 2).

Resistance to early alpha-helical inhibitors was mediated by mutations in the N-terminal heptad repeat region of gp41 [94], providing evidence of the specificity of binding of these peptides to the virus. Monotherapy with enfuvirtide resulted in viral load rebounds after 14 days of therapy, and resistance determinants mapped to the HR1 domain (G36D, I37T, V38A, V38M, N42T, N42D, N43K) [95]. Mutations that confer resistance to enfuvirtide also result in reduced replication capacity / replicative fitness of the virus [96]. This is presumably due to adaptations in the gp41 domain that reduce enfuvirtide binding, but consequently reduce the efficiency of six-helix bundle formation and overall fusion rates [97]. These mutations did not confer cross resistance to other types of entry inhibitors (attachment inhibitors or coreceptor inhibitors) [98] but did sensitize viruses to neutralization by monoclonal antibodies that target the gp41 domain, presumably by prolonging the exposure of fusion intermediates that are specifically sensitive to these antibodies [97]. Adaptation to enfuvirtide has even resulted in viruses that require enfuvirtide for fusion [99].

Though it is clear that gp41 resistance mutations, taken out of the context of the envelope under selection, result in decreased fusion efficiency and reduced viral fitness, the function of envelope context seems to modulate the overall effect of these mutations *in vivo* [100]. Studies of baseline susceptibility to enfuvirtide suggested that large variations in intrinsic susceptibility existed in diverse HIV-1 isolates, and that these variations mapped to regions outside the enfuvirtide binding site [101]. Sequences associated with the V3 loop were correlated with intrinsic enfuvirtide susceptibility, suggesting that interactions with the coreceptor were important determinants of susceptibility of a drug that inhibits virus fusion. A seminal observation in the understanding of entry inhibitor susceptibility was made in 2002 by the discovery that the efficiency of the fusion process was the principal modulator of intrinsic enfuvirtide susceptibility [102]. Mutations in the coreceptor binding site that reduced gp120 affinity for CCR5 resulted in viruses with reduced fusion kinetics [103,104]. Engagement of CD4 by gp120 initiates a process of structural rearrangement in the envelope glycoprotein resulting in fusion. Completion of this process requires engagement of the coreceptor molecule, but enfuvirtide susceptibility is limited to the time between CD4 engagement and six-helix bundle formation. Thus efficiency of this process affects the amount of time that enfuvirtide has to bind gp41 and prevent fusion. Any change in the system that decreases rate of fusion (e.g., reducing the levels of coreceptor expression) also increases susceptibility of virus to inhibition by enfuvirtide. Consistent with this, ENF is synergistic with compounds that inhibit CD4 or coreceptor engagement [105–107].

### 5.3. Chemokine Analogs

Discovery of the chemokine coreceptors was facilitated by the observation that native chemokines can inhibit HIV-1 replication [44]. Thus the first attempt to generate a coreceptor based entry inhibitor was modulation of the endogenous chemokine RANTES (CCL5) to produce a more potent version. Modulation of the N-terminus by deletion of the N-terminal methionine residue resulted in increased potency of the chemokine RANTES [108]. Addition of an aminooxypentane moiety to the N-terminus of either RANTES or MIP-1αP resulted in further increases in potency and increases in affinity for CCR5 [108,109]. Modulation of the N-terminus of the chemokine was specifically chosen because it appears that chemokines interact selectively with specific receptors by a “message” and “address” system. The core domain of the chemokine is specifically responsible for determining receptor specificity (the address), while the N-terminus of the chemokine is involved in elicitation of downstream signal transduction cascades (the message) [110]. An HIV-1 inhibitory molecule should ideally not induce signal transduction cascades or the ensuing lymphocyte activation or chemotaxis. Modifications made to generate met-RANTES and AOP-RANTES resulted in abrogation of chemotaxis signals but not calcium flux [108,111]. This signaling cascade induced by RANTES derivatives can lead to an activation of HIV-1 replication, which can counter the effects of this analog on entry inhibition [108].

One major principal for the development of chemokine-like inhibitors is the ability of chemokines to induce ligand-mediated internalization of their receptor through a clathrin-dependant mechanism. Chemokine receptors, when bound by ligand and in their signaling-active state, activate G protein-coupled receptor kinases (GRKs). GRKs phosphorylate the C-terminal cytoplasmic domain of the chemokine receptor. The phosphorylated form of the receptor is recognized by β-arrestin, which recruits scaffolding proteins and clathrin precursor proteins to the receptor. Clathrin-coated pits form invaginations and the receptor is internalized. CCR5 is specifically endocytosed and moved to a recycling endosomal compartment [112]. Contribution of receptor downregulation in inhibition of HIV-1 entry by chemokines was first observed for inhibition of X4-tropic HIV-1 isolates by SDF-1 [113]. Analysis of a series of RANTES analogs revealed that their potency in blocking HIV-1 entry correlated not with their binding affinity, but with their ability to induce ligand-mediated internalization of CCR5 [114]. Consistent with this, mutagenesis of the CCR5 C-terminus, abrogating its recognition and phosphorylation by GRKs, resulted in a mutant that is not internalized when bound by ligand. Use of this mutant significantly reduced the potency of AOP-MIP-1αP during the course of short term virus exposure (<12hrs) [115]. Beyond simple receptor internalization, it has been found that chemokine analogs result in prolonged sequestration of cell surface receptor [116]. The mechanisms behind prolonged sequestration of CCR5 have been explored but have not been fully elucidated. Further iterative design of the RANTES N-terminus resulted in a third generation compound, PSC-RANTES, with even higher potency than AOP-RANTES [117]. PSC-RANTES has been shown to block vaginal transmission in the SHIV-Macaque model system [118] and is currently in development as a microbicide to prevent sexual transmission of HIV-1.

In some of these macaques treated with high PSC-RANTES concentrations (100μM), a selection of PSC-RANTES resistance variants was confirmed and as described below, resistance was likely conferred by increased coreceptor affinity. In relation to the mechanism of PSC-RANTES inhibition, altered PSC-RANTES or AOP-RANTES sensitivity in primary HIV-1 isolates that remain CCR5-using is likely in the context of either differential binding of virus *versus* drug on the CCR5 receptor, increased affinity of the virus for the receptor, or more rapid entry kinetics. However, CCR5-dependent resistance as opposed to coreceptor switching indicates that the mechanism of PSC-RANTES derivative inhibition cannot be solely due to receptor-ligand internalization or sequestration from the cell surface. Studies suggest that competitive inhibition may even be a dominant mechanism by which PSC-RANTES or other RANTES derivatives block HIV-1 entry [119].

A major problem associated with chemokine analogs is their residual capacity to signal through CCR5 and through cell surface proteoglycans [111,120]. This residual activity has been associated with stimulation of viral replication by enhancement of viral integration [121] and through enhancement of virus entry [122]. AOP-RANTES, like RANTES, has been shown to activate mitogen-activated protein kinases and pertussis-toxin sensitive signals [121,123]. High concentrations of chemokine analogs clearly have a stimulatory effect on T cells, and are formally considered partial agonists of CCR5. This residual signaling activity may limit the effectiveness and potential use of chemokine analogs *in vivo*.

### 5.4. Small Molecule CCR5 Antagonists

Small molecule CCR5 antagonists bind to hydrophobic pockets within the transmembrane helices of CCR5 [124,125]. This site does not overlap the binding sites of either CCR5 agonists or HIV-1 envelope, but instead induces and stabilizes a receptor conformation that is not recognized by either. Thus, these molecules are considered allosteric inhibitors. Ideally, a small molecule inhibitor of CCR5 would block binding by HIV-1 envelope but continue to bind native chemokines and facilitate signal transduction. Most small molecule inhibitors, however, are pure antagonists of the receptor. Oral administration of small molecule antagonists has been demonstrated to inhibit viral replication in macaque models [126] and to prevent vaginal transmission [127]. Thus far, three antagonists (VCV, MVC, and Aplaviroc) have been shown to inhibit virus replication in humans [128]. The compound maraviroc (MVC) has been tested in phase III efficacy trials and was approved for therapeutic use by the FDA in 2007.

Maraviroc is a selective small molecule CCR5 antagonist currently being used to treat patients with resistance to multiple HIV drugs but has been recently approved for first-line treatment regimens. Identified in a chemokine binding assay screen of a Pfizer compound library, maraviroc is an imidazopyridine that binds a hydrophobic transmembrane cavity of CCR5. Alanine scanning mutagenesis of the transmembrane domains of CCR5 revealed key residues in domains 1, 2, 3 and 7 for small molecule binding [124]. Binding alters the conformation of the second extracellular loop of the receptor, preventing its interaction with the V3 stem loop of HIV’s gp120 envelope glycoprotein [124,129]. Ligand binding competition assays indicated maraviroc prevented C-C chemokine ligand binding to CCR5 with IC_50_ values (concentration of drug required to inhibit viral replication by 50% in culture) in the nanomolar range. Additionally, it inhibited chemokine-induced signaling of intracellular calcium redistribution, however it did not trigger calcium signaling upon binding up to 10μM [128]. In the same study, maraviroc was shown to have potent antiviral activity against a panel of primary CCR5-tropic HIV-1 isolates with a mean IC_90_ of 2 nM.

The MOTIVATE 1 (conducted in North America) and MOTIVATE 2 (conducted in Europe, Australia, and the US) phase III clinical trials sought to study the safety and efficacy of maraviroc in multi-drug resistant patients harboring R5 tropic viruses. (MOTIVATE stands for Maraviroc *versus* Optimized Therapy In Viremic Antiretroviral Treatment Experienced patients) Results from these trials published in 2008 indicated patients receiving maraviroc (either once or twice daily) plus an optimized background therapy (OBT) showed significant reductions in HIV-1 RNA levels and increases in CD4 cell counts *versus* patients receiving OBT alone [130,131]. Preliminary results from these trials resulted in FDA approval of maraviroc for clinical use in 2007. It is currently marketed as SELZENTRY by Pfizer, Inc. Since its approval, maraviroc has been utilized as a salvage therapy for multi-drug resistant patients with R5 tropic virus.

Vicriviroc is another CCR5 inhibitor by Schering Plough currently in phase III clinical trials that binds a similar transmembrane pocket as maraviroc. Preliminary results from the vicriviroc clinical trials indicate a reduction in viral load in patients taking vicriviroc in combination therapy [132]. In addition to maraviroc and vicriviroc, several other small molecule inhibitors are in various stages of development (Table 2). Aplaviroc was developed as a small molecule CCR5 antagonist to block HIV-1 entry but GlaxoSmithKline halted development in a phase II trial due to strong evidence of hepatic toxicity [133].

## 6. Resistance to Coreceptor Inhibitors

**Figure 3.**
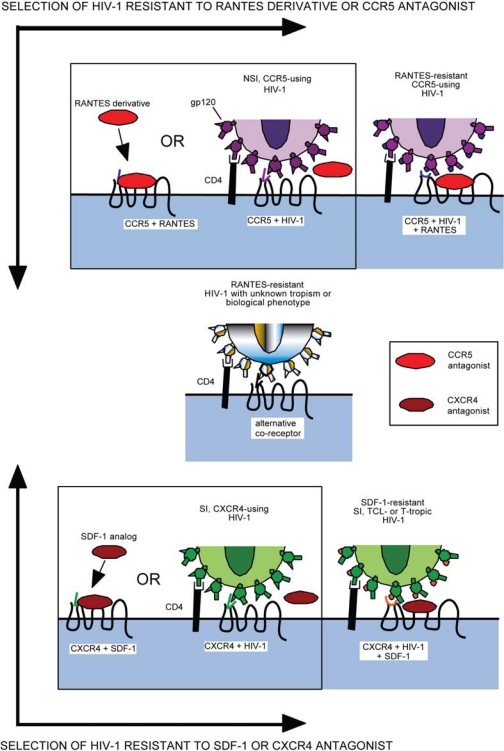
Schematic of HIV-1 resistance to co-receptor inhibitors based on putative co-receptor switch or binding of the drug bound inhibitor. Resistance to CCR5 inhibitors (top) or CXCR4 inhibitors (bottom) can manifest as a change in co-receptor tropism (center) or the virus acquiring the ability to utilize inhibitor-bound forms of the co-receptors (right top and bottom).

As with any antiretroviral, resistance to entry inhibitors will inevitably arise. However, their resistance profile will be unlike any other class of antiretroviral since they bind to a host cell protein, not a viral protein. Potential mechanisms of resistance could include 1) tropism switching (utilization of CXCR4 instead of CCR5 for entry), 2) increased affinity for the coreceptor, 3) utilization of inhibitor-bound receptor for entry, and 4) faster rate of the entry step. A general schematic of HIV-1 inhibition by and resistance to a CCR5 or CXCR4 agonist/antagonist is provided in Figure 3.

### 6.1. Tropism Switching

The major concern in the therapeutic administration of coreceptor inhibitors is the possibility that resistance will manifest by a change in coreceptor tropism from CCR5 to CXCR4, or that an outgrowth of an X4-tropic virus subset will come to dominate the intrapatient virus population. The mechanisms and consequences of coreceptor switching are poorly understood at this time. It is unclear what factors drive this switch, or why switching is generally limited to late phases of disease progression. Indeed, it is not clear at this time whether a switch from R5 to X4 tropism is a cause or consequence of disease progression.

On the other hand, resistance to a variety of small molecule CCR5 inhibitors has been generated by passage of inhibitor-susceptible virus isolates in sequential dose escalations of drug. These propagations have been performed in PBMC cultures, which express CCR5 and CXCR4, as well as a variety of other chemokine receptors that have been previously implicated as potential HIV-1 coreceptors. In these experiments, inhibitor-resistant viruses continue to require CCR5 for entry and cannot utilize either CXCR4 or any alternative coreceptors [134,134–137] (Figure 3). Furthermore, evaluation of coreceptor tropism of viruses derived from patients who failed maraviroc therapy during the MOTIVATE trials indicate that tropism changes occurred only when X4 tropic viruses were pre-existing in the patient quasispecies [138]. Of 1042 patients with R5 tropic virus at the time of enrollment, 228 patients reportedly failed treatment. Of these failures, 133 were receiving maraviroc and of these 76 patients were found to harbor dual or mixed tropic virus at the time of failure [131]. Tropism testing revealed that these patients had pre-existing populations of X4-tropic virus prior to the start of the trial, which was below the level of detection of the tropism assay used at the time of enrollment. Another trial specifically studying effects of maraviroc on patients with non-R5 tropic virus found no benefit in maraviroc treatment *versus* placebo [139]. Thus it appears that, though outgrowth of pre-existing X4-tropic viruses in the quasispecies remains a problem for the therapeutic administration of CCR5 antagonists, *de novo* mutations conferring altered coreceptor usage may not be the favored pathway for resistance *in vitro* or *in vivo*.

It is important to note that a coreceptor switch or evidence of pre-existing CXCR4 tropic virus only provide a reason for 76 of 133 MVC failures. As described below, four of the MVC treatment failures may relate to use of CCR5 even in the presence of MVC. However, 55 MVC treatment failures remain unexplained at the time of this publication. Recent studies suggest a possible discrepancy between differential sensitivity to CCR5 antagonists in various phenotypic assays. For example, susceptibility to entry inhibitors, specifically CCR5 antagonists, can be modulated dependent on cell type, state of cellular activation, and number of virus replication cycles [119,134,136,140]. Also, different primary HIV-1 isolates can vary in sensitivity to entry inhibitors as much as 100-fold in IC_50_ values [119,128]. This difference is much more demonstrable with infection assays using replication competent primary HIV-1 isolates as compared with defective viruses limited to single cycle replication. In 2005, Dorr *et al.* reported less than a 10-fold variation in MVC sensitivity with envelope pseudotyped viruses. In contrast, primary isolates in multiple cycle assays displayed a 100-fold variation in MVC sensitivity but only a 10-fold inhibition difference in single cycle assays [128]. Thus, it is quite possible that the remaining 55 failures in the MOTIVATE trials might relate to sensitivity of the infecting virus to MVC prior to treatment or simply, the inability to detect MVC resistance at the time of treatment failure with existing assays.

### 6.2. Competitive Resistance Model: Increased Coreceptor Affinity

**Figure 4.**
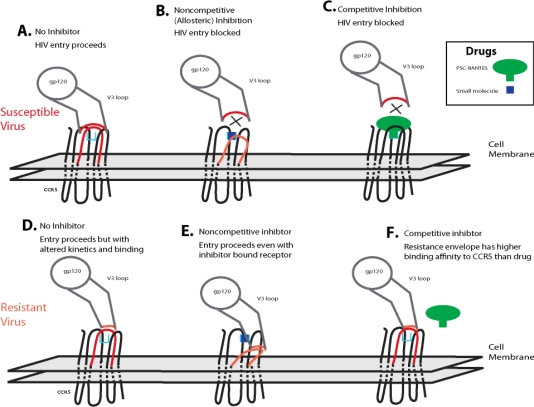

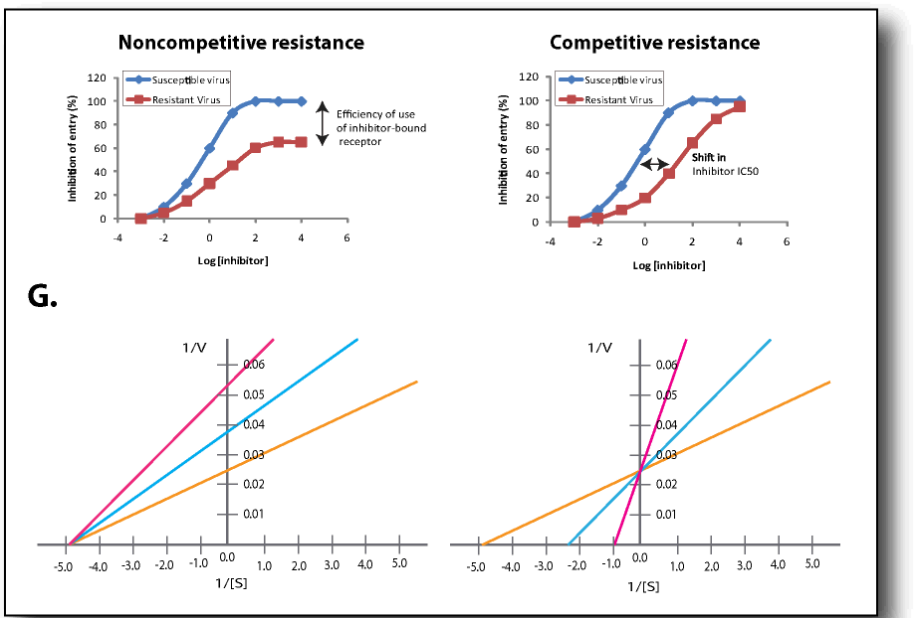
Models of Resistance to HIV-1 Entry Inhibitors. Two models for resistance to HIV-1 coreceptor inhibitors have been proposed. The noncompetitive model has been proposed for resistance to small molecule allosteric inhibitors of CCR5 (such as maraviroc, vicriviroc and TAK-779). In this model, **(b)** envelopes that are sensitive to inhibition cannot recognize the configuration of the inhibitor bound receptor. **(e)** Inhibitor-resistant variants can recognize the inhibitor-bound receptor complex. **(g)** Entry inhibition assays result in a plateau effect, and the height of this plateau is indicative of the efficiency with which the HIV-1 envelope utilizes the inhibitor-bound form of CCR5 relative to the inhibitor-free receptor (a virus that cannot use the inhibitor-bound complex is indicated in blue diamonds, while a virus that uses the inhibitor-bound form of CCR5 with 35% efficiency as compared to the inhibitor-free receptor is indicated in red squares). In this model the maximum rate of the reaction (1/V) reduces in the presence of inhibitor without changing the binding affinity (1/[S]) of the substrate for the receptor. The competitive model may be the most relevant for inhibitors that block HIV-1 envelope binding by steric hindrance (chemokines, antibodies to CCR5). In this model, **(c)** an inhibitor bound receptor is not accessible by HIV-1 envelope proteins with low overall affinities for receptor, **(f)** whereas envelope proteins with mutations that confer high affinity can compete off the inhibitor for interaction with the receptor. **(g)** This resistance mechanism is manifested in entry inhibition assays by a fold change in IC_50_ value (sensitive virus is depicted by blue diamonds, while a resistant variant is depicted by red squares). In this model, increasing inhibitor concentrations result in successful inhibition (100%) of entry. The affinity of the substrate to the binding site is decreased while the maximum velocity of the reaction remains unchanged.

If coreceptor switching is not the main mechanism of resistance to small molecule coreceptor inhibitors, then it is important to understand by what mechanism resistance does arise. Two models of resistance have been proposed: competitive and noncompetitive resistance (Figure 4). In the competitive resistance model, gp120 interacts with CCR5 with a given affinity. In the presence of an inhibitor (green), the high affinity interaction between receptor and inhibitor blocks the lower affinity interaction between the receptor and gp120 (Figure 4c). Adaptations in gp120 increase the affinity relationship between envelope and coreceptor, and this change in receptor affinity is sufficient for gp120 to compete inhibitor off of the receptor (Figure 4f). Experimental evaluation of this resistance mechanism would appear as a shift in IC_50_ value and complete inhibition of virus entry can be achieved by increasing the concentration of inhibitor (Figure 4g). Many questions about this potential form of resistance can be raised: 1) what are the baseline affinities of each contact point between gp120 and coreceptor? 2) How great are the variations among HIV-1 isolated in these baseline affinities? 3) What types of change in the gp160 sequence would result in greater coreceptor affinity? 4) How great a change in affinity is required to result in differential inhibition by a given drug? 5) Will gp120 adapt to different CCR5 inhibitors by a similar pathway, or by alternative mutations for each drug (*i.e.,* will cross resistance be a problem)? 6) What are the consequences of affinity changes on overall viral replication? 7) What are the consequences of affinity changes on viral dynamics *in vivo* and disease progression? Much work is still required to substantially address these issues.

### 6.3. Non-competitive Resistance Model: Utilization of Inhibitor-bound Coreceptor

The second model of resistance to coreceptor inhibitors is noncompetitive resistance (Figure 4). In this scenario, a small molecule inhibitor binds CCR5 in an allosteric site, which induces and/or stabilizes a receptor configuration that is not recognized by HIV-1 envelope (or native chemokines). Due to the lack of recognition, receptor docking is blocked and the HIV-1 entry process cannot proceed (Figure 4b). Adaptation to this type of inhibition is mediated by changes in gp120 that allow for recognition of the CCR5 configuration induced by inhibitor binding in the allosteric site. The overall affinity for the inhibitor-free receptor may not be relevant in this circumstance, and the envelope can complex with the inhibitor-bound configuration of CCR5 (Figure 4e). Experimental evaluation of noncompetitive resistance is manifested by a plateau effect on HIV-1 entry as inhibitor concentration increases (Figure 4g). In this case, the overall extent of inhibition never reaches 100% independent of the concentration of inhibitor. Evaluation of inhibition curves would reveal that the EC_50_ for an inhibitor resistant virus (defined as the half-plateau height, or the concentration at which the inhibitor achieves 50% of its maximal effect) is equal to the IC_50_ of the inhibitor against the inhibitor-sensitive variant of the virus. The plateau effect is caused by differences in the efficiency of usage of the inhibitor-free and inhibitor-bound form of the receptor. Thus a completely sensitive virus uses the inhibitor-bound form of CCR5 with 0% efficiency. A virus that uses the inhibitor-bound form of CCR5 with equal efficiency as the inhibitor-free form of CCR5 would not indicate any level of inhibition. Consequently, any virus adaptation that would cause an increase in the efficiency of use of the inhibitor-bound receptor over the efficiency of use of the inhibitor-free receptor would yield a negative inhibitory value, or an apparent enhancement of infection in the presence of saturating levels of inhibitor. This has led to redefining phenotypic resistance as a reduction in the maximal percent inhibition (MPI) <95% rather than a classical shift in IC_50_ values. The pattern of mutations in *env* conferring this drug resistant phenotype, *i.e.*, entry through drug-bound receptor, is largely unknown but could be related to V3 loop mutations (Table 3). *In vivo*, this resistance mechanism has been observed in several studies. For example, an HIV-1 subtype C infected patient treated with vicriviroc failed treatment and was shown to harbor virus capable of replicating even at the highest drug concentration (1μM) [141]. With this patient’s HIV-1 envelope, there was no apparent shift in IC_50_ values to VCV. Resistance to PSC-RANTES was observed in macaque microbicide studies and the SHIV *env* variant was capable of replicating even at 100 nM of drug, a concentration 100-fold above the IC_50_ for wild type SHIV *env* [142]. In contrast to the observation with VCV resistance, this PSC-RANTES resistant virus displayed a shift in drug susceptibility curve suggesting that increased binding affinity for native CCR5 and displacement of PSC-RANTES *versus* PSC-RANTES bound CCR5 may be more dominant in the drug resistant phenotype. This combination in mechanisms has also been observed in resistant HIV-1 isolates selected under VCV selective pressure *in vitro* [134].

During the MOTIVATE clinical trials, several patients failed treatment yet maintained CCR5 tropism. While some of these patients experienced treatment failure as a result of opportunistic infections, a subset experienced failure as a result of apparent resistance to maraviroc. Clonal analyses of the *env* regions of viruses from these patients indicated changes had occurred in the V3 loop of gp120 as compared to viruses isolated at the time of enrollment. However, the pattern of mutations was different in each patient implying multiple pathways to resistance were possible [143].

Currently, there is limited data available from *in vitro* resistance studies. However, a few studies have described *in vitro* generated resistant variants of primary HIV-1 isolates. In 2006 maraviroc resistant variants were generated from two different primary HIV-1 isolates, RU570 (clade G) and CC1/85 (clade B), by sequential passage in the presence of maraviroc [136]. Both resistant viruses possessed distinct amino acid changes in the V3 loop of gp120 as compared to control virus passages (Table 3). For CC1/85_res_ virus A316T and I323V mutations were observed in the V3 loop while the RU570_res_ virus had a three amino acid (QAI) deletion from residues 315–317. Both resistant viruses had additional mutations in other regions of *env* outside of V3 but site-directed mutagenesis studies of molecular clones created from CC1/85_res_ indicated that the V3 loop changes were responsible for conferring maraviroc resistance. The reduction in MPI observed for the resistant viruses indicated these viruses were able to utilize inhibitor-bound receptor for entry. To further support this hypothesis, maraviroc resistant viruses that were susceptible to aplaviroc (a CCR5 inhibitor that binds to a similar region as maraviroc) were unable to be inhibited by aplaviroc in the presence of high concentrations of maraviroc.

Studies describing other resistant primary isolates vary widely in adaptive amino acid changes observed in the envelope proteins. Genotypic and phenotypic analysis of CC1/85 passage variants that ultimately gave rise to resistance to AD101 indicated that resistance arose via stepwise accumulation of mutations in the V3 region of gp120 [140]. A single mutation was selected for in V3 after four to six passages in the presence of AD101 that resulted in modest resistance (three-fold) through an apparent competitive mechanism. This variant had increased capacity to utilize low levels of CCR5 [135] and is hypothesized to have greater overall CCR5 affinity. Upon continued passage, three more mutations arose in the V3 which led to complete insensitivity to AD101. Inhibition studies of virus clones bearing these four mutations resulted in a plateau effect which indicated the virus was using the inhibitor-bound CCR5 complex with approximately 90% efficiency of the inhibitor-free form of CCR5, though cell type differences in plateau height did exist [144]. However, adaptation to vicriviroc of this same primary isolate did not involve changes in the V3 region, suggesting that resistance can arise through alterations in envelope geometry that are not restricted to specific sites within the envelope glycoprotein [134,145]. These studies suggest that viruses from the same primary isolate can follow multiple genetic pathways to resistance.

Additional reports of chemokine receptor directed small molecule resistant viruses have largely implicated mutations in the V3 region as necessary and sufficient for resistance. Mutations in the V3 loop sequence of virus from the subtype C infected individual harboring VCV resistant virus were found sufficient to confer vicriviroc resistance [141]. More specifically the emergence of the S306P mutation was associated with complete resistance to vicriviroc in chimeric clones. Utilizing the clade G primary isolate RU570, a vicriviroc resistant variant was isolated and mutations in the V3 loop and C4 region were identified as responsible for conferring resistance [146]. Resistance to TAK-652 manifested as changes in the V3 loop as well as other regions of gp160 [137]. A single K315R mutation in the V3 loop of SHIV_SF162-p3_ conferred at least 10-fold resistance to PSC-RANTES, which was selected under a single dose of drug applied as a microbicide [142]. More importantly, virus harboring the K315R mutation was still capable of replicating in the presence of 100 nM PSC-RANTES or a concentration at least 100-fold above the IC_50_ value for the wild type virus. Ultimately, the changes required in gp120 to allow for recognition of inhibitor-bound forms of CCR5 are unclear, and the alterations in the geometry of the envelope – receptor complex remain a mystery.

Of note is that several of the studies describing generation of resistant viruses have utilized primary isolate CC1/85 or a derivative clone to generate resistance to maraviroc, vicriviroc, or AD101 [134,140,147]. This parental virus was derived from a subtype B individual in 1985 that had viruses only able to use CCR5. Use of this single primary isolate has resulted in multiple sets of mutations conferring resistance to each of these drugs implying multiple pathways of resistance can be followed by the same virus. Only two studies have described resistance developed in a non-subtype B virus (clade G RU570) against maraviroc and vicriviroc [136,146]. There is a clear need for more studies characterizing small molecule resistance in non-subtype B isolates.

Cross-resistance has been described for some but not all of the *in vitro* derived resistant variants. Two maraviroc resistant variants remained susceptible to other small molecule CCR5 antagonists and the fusion inhibitor enfurvitide [136]. In contrast, vicriviroc resistant variants acquired resistance to the modified chemokine derivatives PSC-RANTES and AOP-RANTES as well as other small molecule CCR5 inhibitors, namely AD101, SCH-C, and CMPD 167 [134]. An AD101 resistant variant acquired resistance to the small molecule SCH-C yet remained susceptible to inhibition with the chemokine ligand RANTES [140]. Therefore the implications of the development of resistance to one small molecule CCR5 inhibitor to cross-resistance to other members of this class are not yet clear.

In summary, mutations associated with resistance to CCR5 antagonists in *in vitro* derived resistant isolates have largely mapped to the V3 region of gp120 with the exception of two viruses with resistant mutations in other regions of gp160. However, resistance in clinical trial patients failing treatment with R5 tropic viruses have all been attributed to mutations in the V3 loop arising during therapy [141,143]. While cross-resistance has been reported for some resistant variants, others have remained susceptible to small molecule inhibitors.

## 7. Intrinsic Resistance to Entry Inhibitors

Primary HIV-1 isolates display a wide range of susceptibilities to entry inhibitors, with 50% inhibitory concentrations (IC_50_) varying by as much as 1000-fold. This is in significant contrast to inhibitors of reverse transcription and protease cleavage, which exhibit modest differences in intrinsic sensitivity across diverse HIV-1 isolates. Large susceptibility variations in primary HIV-1 isolates have been documented for the chemokine derivative AOP-RANTES [148], the fusion inhibitor enfuvirtide (ENF, T-20) [102,103,149], and many small molecule coreceptor antagonists [TAK-779 [102,150], maraviroc [128], SCH-C [150], vicriviroc [151], and AMD-3100 [149]. Due to the high degree of diversity among HIV-1 *env* genes of the same or different HIV-1 subtypes, it is difficult to identify specific sequence variations that may be associated with variable sensitivity to entry inhibitors.

Evaluation of intrinsic sensitivity differences to T-20 and TAK-779 revealed that kinetic factors of fusion were largely responsible for variations in IC_50_ [102]. Sensitivity to T-20 mapped to the V3 loop of *env* [101], but mutations in the bridging sheet are also sufficient to modulate intrinsic susceptibility to these inhibitors [102,103]. Multiple factors are involved in the efficiency of host cell entry. Upon CD4 binding, structural rearrangements within the envelope occur which reveal the coreceptor binding site. The current model of ternary complex formation favors multiple interaction sites between HIV-1 envelope and CCR5. The bridging sheet and V3 stem interact with the CCR5 N-terminus, while the V3 crown interacts with the second extracellular loop of CCR5. The hypervariable V3 loop must evolve by balancing attempts to escape host humoral response with the need to engage the CCR5 coreceptor for host cell entry. The affinity relationship between CCR5 and envelope, which can be modulated by the density of CCR5 on the cell surface, may be important in influencing the efficiency of entry. Some views hold that the major rate-limiting process in host cell entry is the formation of six-helix bundles [152], but other data suggests that ternary complex formation is the major rate-limiting step of the entry process [153]. The affinity relationship between CCR5 and V3 may be important in influencing the efficiency of entry through either of these pathways.

Mechanisms involved in variable susceptibility to chemokines such as RANTES or their derivatives have not been evaluated. These inhibitors differ from small molecule CCR5 antagonists in their ability to occupy surface receptor as well as trigger internalization of CCR5 [114]. A >30-fold difference in intrinsic susceptibility to the CCL5 analog AOP-RANTES was observed with a panel of primary HIV-1 isolates from all subtypes [148]. This variability in AOP-RANTES sensitivity may be related to sequence differences in the V3 crown, specifically at positions 318 and 319 (HXB2 numbering). Similar variations at position 319 were observed when comparing intrinsic sensitivities of a different panel of primary isolate viruses to inhibition by TAK-779 and SCH-C [150]. Further underscoring the potential relevance of these sites in intrinsic entry inhibitor sensitivity, treatment of HIV-infected hu-PBL-SCID mice with the CCL5 analog NNY-RANTES selected for mutation at position 318 in the challenge virus, suggesting a potential role of this polymorphism in escape [154].

Subsequent studies revealed that the most common polymorphisms at positions 318 and 319 (e.g., 318Y and 319A) in the V3 loop showed the greatest intrinsic resistance to not only RANTES derivatives but also inhibition by enfuvirtide and TAK-779 [155]. In contrast, the more rare polymorphisms (but still found as wild type sequence) such as 318R and 319T were hypersusceptible to these entry inhibitors. These findings also revealed that the more common polymorphisms in HIV-1 *env* may have evolved to increase replicative fitness, escape neutralizing antibodies, and avoid the inhibitory effects of CC-chemokines such as CCL3 (or RANTES). As a consequence of selected evolution, many primary HIV-1 isolates may be intrinsically resistant. However, this intrinsic resistance is highly context dependent. For example, substituting just the V3 loop coding region of a more sensitive primary HIV-1 isolate into a laboratory strain (more resistant) backbone resulted in demonstrable decrease in susceptibility to all entry inhibitors. Sensitivity to entry inhibitors increased when large segments of the *env* gene of primary HIV-1 isolates were substituted into the laboratory strain or when the primary isolates themselves were employed for drug susceptibility assays.

Variations in the efficiency of the entry process may have an important role in the context of inhibiting HIV-1 entry *in vivo*. Many studies have looked at the baseline susceptibility of primary isolate HIV-1 viruses to inhibition by a broad range of entry inhibitors. As was seen with enfuvirtide, a wide range of susceptibility (>1000-fold) exists among primary HIV-1 isolates to molecules that inhibit at the level of the coreceptor [101,148–151,156]. The clinical relevance of such variations is unclear at this time. Furthermore, the mechanisms involved in these variations are not clear. The intrapatient population of viruses must infect cells in the presence of chemokine inhibitors, and some studies have suggested that viruses grow resistant to inhibition by chemokines over the course of HIV-1 infection in a single patient [157]. One possible consequence of this hypothesis is that virus variants found at acute infection may be more susceptible to entry inhibitors than variants found late in infection. Variations in coreceptor affinity may be involved in determining sensitivity to coreceptor inhibitors.

## 8. Implications of HIV-1 Entry Efficiency

HIV-1 envelope-mediated entry is a highly cooperative process. The efficiency of this process can be modulated by factors specific to the host cell, including receptor density on the surface, as well as expression of receptors in antigenically permissive conformations. Entry efficiencies are further modulated by the HIV-1 envelope both through quantitative factors such as receptor affinity as well as through qualitative factors of receptor interaction (e.g., envelope geometry and receptor-envelope stoichiometry).

Several approaches have been taken to assess cooperativity in HIV-1 envelope mediated membrane fusion. The earliest study evaluated the effect of varying CCR5 expression levels on virus infection, and estimated that six CCR5 molecules were involved in a stable entry complex [158]. By titrating out functional envelope trimers using a dominant negative gp160 variant, it has been suggested that HIV-1 entry can be achieved by a single trimer [159], and further that two subunits of the trimer are sufficient for entry [160]. This suggests that two CD4 molecules and two CCR5 molecules would be involved in this process. However, previous studies have implicated a role for at least four CD4 interactions in entry [161], and similar dominant negative gp160 approaches have suggested the involvement of between four and five trimers [162].

There are several implications to the multiple receptor stoichiometry of HIV-1 entry. One assumption is that receptor density will play a significant role in entry efficiency, and furthermore, affinity relationships between envelope and CD4 as well as envelope and coreceptor will have implications on the rate and efficiency of entry. A stoichiometry requiring the aggregation of multiple CCR5 molecules to form a stable entry complex will be significantly inhibited in environments with limiting amounts of CCR5, such as in the presence of inhibitor. This prolongation of the entry step may allow for greater efficacy of various virus deactivation processes, such as endocytosis or complement mediated lysis.

The efficiency of the HIV-1 entry process is thought to have implications for overall viral replicative fitness [163,164]. HIV-1 exists as a genetically variable population, or quasispecies [165,166] as a consequence of a high mutation rate (3.4 × 10^−5^ per base pair) [1] and rapid turnover. Fitness is a complex factor within an infected host. In a simple model, intrinsic rates of viral replication determine the relative proportion of an HIV-1 clone in the overall population. Although low fitness variants can contribute to the genetic pool in the quasispecies theory, the most fit variants will dominate the population due to their competitive replicative advantage. Host selection is a major determinant of fitness *in vivo*. However, replicative fitness, also termed replication capacity (a measure of the growth kinetics of a virus) has important implications on viral fitness and pathogenesis. For example, viruses derived from long term nonprogressor cohorts have been shown to have lower replicative fitness than viruses from individuals with normal disease progression [167]. Viruses lacking the *nef* gene also have a replication defect *in vitro*, and these viruses are frequently associated with less severe disease symptoms. Efficiency of virus entry may be an important determinant of viral replicative fitness, and consequently of viral pathogenesis.

In the case of primary HIV-1 isolates, there is a direct relationship between replicative fitness and sensitivity to many entry inhibitors. As described earlier, primary HIV-1 isolates can evolve to higher replicative fitness in possible relation to avoidance of host immune response, higher cell tropism/entry efficiency, and possible avoidance of β chemokine inhibition [148,155,164]. These same selective pressures could result in viruses with increased binding affinity to co-receptor and higher rate of entry kinetics and as a consequence outcompete inhibitor or simply utilize inhibitor-bound CCR5 receptor. To support this hypothesis, HIV-1 envelopes derived from patients with elite suppression displayed reduced entry efficiencies, slow entry kinetics, and poor CCR5 utilization [168]. There was also a trend for these “elite” envelopes to be more sensitive to entry inhibitors. Lobritz *et al.* (2007) described a strong correlation between the replicative fitness and entry inhibitor sensitivity of HIV-1 harboring natural V3 polymorphisms [155]. This correlation was observed with PSC-RANTES, ENF, and the 2D7 neutralizing antibody but not with TAK-779. The latter three drugs likely inhibit through a competitive mechanism whereas TAK-779 may be more of an allosteric, noncompetitive inhibitor. As a consequence, primary HIV-1 isolates are unlikely to evolve to utilize a coreceptor bound to a synthetic drug without a natural analog. Thus, resistance to drugs such as VCV or MCV might follow more classical selection of drug resistance and result in a fitness decrease.

Very few studies have explored the fitness impact of HIV-1 with acquired resistance to CCR5 agonist or antagonist and none have explored the HIV-1 fitness effects with actual MVC or VCV treatment of patients and emergence of resistance. However, two *in vitro* studies suggest that a decrease in fitness resulting from CCR5 inhibitor resistance may not be as common as with the fitness loss observed with resistance to almost all other antiretroviral drugs. Anastassopoulou *et al.* (2007) indicated that VCV-resistant variants selected *in vitro* were actually more fit or of similar fitness than the parental HIV-1 isolate [147]. In vaginal microbicide trials in macaques, PSC-RANTES selected for SHIVenv viruses with a single V3 and gp41 mutation that conferred resistance but actually increased replicative fitness over the infecting virus by at least 100-fold [142]. This increase in fitness was even greater when utilizing rhesus macaque CCR5 than human CCR5. Together these findings suggest that a major mechanism for resistance to CCR5 antagonists/agonists may relate to increased affinity for CCR5 and increased entry efficiency in the presence or absence of the drug.

## 9. Conclusions

Clearly many questions remain in our understanding of HIV-1 entry. It has become essential to answer many of these questions as inhibitors of the HIV-1 entry process begin widespread use in the treatment of HIV infection. The discovery and successful development of CCR5 entry inhibitors proves viral entry is a viable target for therapeutic intervention in HIV-1 patients. The only FDA approved CCR5 antagonist, maraviroc, has been utilized as salvage therapy for HIV patients harboring multi-drug resistant viruses and has recently been approved as therapy in treatment naïve patients. The propensity of HIV to develop drug resistance however, will ultimately result in treatment failures to this new class of inhibitors. Therefore it is of upmost importance to understand how resistance develops and by what mechanism(s) the virus is able to subvert inhibition. The research highlighted here provides a starting point towards understanding the complex mechanisms of resistance to HIV-1 entry inhibitors.

## Figures and Tables

**Table 1.** FDA approved therapeutics for the treatment of HIV infection [3]. NRTI – nucleoside reverse transcriptase inhibitor; NNRTI – non-nucleoside reverse transcriptase inhibitor; PI – protease inhibitor.

**Brand Name**	**Generic Name**	**Class**	**Manufacturer**	**Approval Date**
Emtriva	emtricitabine, FTC	NRTI	Gilead Sciences	07/02/03
Epivir	Lamivudine, 3TC	NRTI	GlaxoSmithKline	11/17/95
Hivid	Zalcitabine, ddC	NRTI	Hoffmann-LaRoche	06/19/92
Retrovir	Zidovudine, AZT	NRTI	GlaxoSmithKline	03/19/87
Videx	Didanosine, ddI	NRTI	Bristol-Myers Squibb	10/09/91
Viread	Tenofovir, TDF	NRTI	Gilead Sciences	10/26/01
Zerit	Stavudine, d4T	NRTI	Bristol-Myers Squibb	06/24/94
Ziagen	Abacavir, ABC	NRTI	GlaxoSmithKline	12/17/98
Intelence	Etravirine, ETV	NNRTI	Tibotec Therapeutics	01/18/08
Rescriptor	Delavirdine, DLV	NNRTI	Pfizer	04/04/97
Sustiva	Efavirenz, EVF	NNRTI	Bristol-Myers Squibb	09/17/98
Viramune	Nevirapine, NVP	NNRTI	Boehringer Ingelheim	06/21/96
Agenerase	Amprenavir, APV	PI	GlaxoSmithKline	04/15/99
Aptivus	Tipranavir, TPV	PI	Boehringer Ingelheim	06/22/05
Crixivan	Indinavir, IDV	PI	Merck	03/13/96
Invirase	Saquinavir, SQV	PI	Hoffmann-LaRoche	12/06/95
Lexiva	Fosamprenavir, APV	PI	GlaxoSmithKline	10/20/03
Norvir	Ritonavir, RTV	PI	Abbott Laboratories	03/01/96
Prezista	Darunavir	PI	Tibotec, Inc.	06/23/06
Reyataz	Atazanavir, ATV	PI	Bristol-Myers Squibb	06/20/03
Viracept	Nelfinavir, NFV	PI	Agouron	03/14/97
Fuzeon	Enfuvirtide, T-20	Fusion	Hoffman-LaRoche and Trimeris	03/13/03
Selzentry	Maraviroc, MVC	CCR5/entry	Pfizer	08/06/07
Isentress	Raltegravir, RTV	Integrase	Merck	10/12/07

**Table 2.** Investigational Entry Inhibitors.

**Compound**	**Mechanism**	**Status**	**Manufacturer**
T-20	Fusion inhibitor	Approved	Trimeris
T-1249	Fusion inhibitor	Discontinued	Trimeris
C-34	Fusion inhibitor	Preclinical only	---
5-Helix	Fusion inhibitor	---	---
Maraviroc	CCR5 antagonist	Approved	Pfizer
Vicriviroc (SCH-D)	CCR5 antagonist	Phase III	Schering-Plough
SCH-C	CCR5 antagonist	Phase I	Schering-Plough
AD101	CCR5 antagonist	Preclinical only	Schering-Plough
Aplaviroc	CCR5 antagonist	Discontinued from Phase I	GlaxoSmithKline
TAK-652	CCR5 antagonist	Phase I	Takeda
TAK-779	CCR5 antagonist	Preclinical only	Takeda
INCB 9471	CCR5 antagonist	Phase IIa	Incyte
AMD3100	CXCR4 antagonist	Phase I/II	AnorMED
PRO-140	Humanized anti-CCR5 monoconal antibody	Phase IIa	Progenics
PSC-RANTES	Chemokine analog	(Microbicide)	Gryphon
BMS-378806	Attachment inhibitor	Phase IIa	Bristol-Myers Squibb
PRO-542	CD4-Ig fusion	Discontinued	Progenics
TNX-355	Anti-CD4 monoclonal antibody	Phase IIa	Biogen Idec; Tanox

**Table 3.** V3 loop resistance mutations of CCR5 small molecule resistant viruses.

**Resistant virus**	**V3 Loop resistance mutations (HXB2 numbering)**	**Reference**
CC1/85 – Maraviroc	316T 323V	[136]
RU570 – Maraviroc	QAI deletion 315–17	[136]
RU570 - Vicriviroc	305R 315Q 319T	[146]
D101.12-Vicriviroc (CC1/85 derived)	308P	[134]
CC101.19 – AD101 (CC1/85 derived)	305R 308P 316V 321E	[140]
Week 28 *in vivo* patient isolate- Vicriviroc	305R 306P 307I 316I 318R 319E	[141]
